# Megaprosthetic replacement in complex distal humerus fractures in elderly patients: a case series

**DOI:** 10.1186/s13018-023-04465-2

**Published:** 2024-01-11

**Authors:** Matteo Caredda, Luigi Cianni, Andrea De Fazio, Antonio Ziranu, Raffaele Vitiello, Giulio Maccauro

**Affiliations:** 1 Department of Ageing, Neurosciences, Head-Neck and Orthopedics Sciences, Orthopedics and Trauma Surgery, Fondazione Policlinico Universitario Agostino Gemelli IRCCS, 00168 Rome, Italy; 2 Orthopedics and Trauma Surgery, Università Cattolica del Sacro Cuore, 00168 Rome, Italy

**Keywords:** Megaprostheses, Megaprosthesis, Elbow, Elderly, Elbow replacement, Distal humerus, Distal humerus fractures

## Abstract

**Background:**

Managing distal humeral fractures can be challenging for orthopedic surgeons. There are several treatment options for managing this type of fracture, and the treatment method for these fractures should be based on patient-related factors. In elderly patients with osteoporotic bone and severe comminution of the fracture, adequate fixation can be a major challenge for surgeons. The use of megaprosthesis has been recently proposed in traumatology as an alternative to osteosynthesis or conventional prosthesis for the management of comminuted articular fractures in elderly patients with poor bone stock.

**Methods:**

A consecutive case series of 5 patients who underwent reconstruction of the elbow joint with a trabecular hinged modular elbow megaprosthesis was reviewed retrospectively. All patients included had AO/OTA 13C2 and 13C3 fractures with metaphyseal extension and considerable bone loss of the distal humerus. The primary outcome was the evaluation of functional and clinical outcomes with the MEPS score in comminuted distal humerus fractures with metaphyseal extension and poor bone stock in elderly patients treated with elbow megaprosthesis. The secondary outcome was assessing the treatment-related complication rate of this technique in non-oncological fields.

**Results:**

Five patients were included in the study with a mean age of 82.66 ± 7.72 years at surgery. The mean MEPS value was 63 ± 24.2 at 1 month, 81 ± 23.53 at 3 months, 83 ± 24.2 at 6 months, and 84 ± 24.57 at 12 months. No intraoperative complications were recorded in our series. Of 5 patients, four patients had excellent clinical and functional outcomes. We did not encounter wound dehiscence, prosthetic joint infection, aseptic loosening, or periprosthetic fractures.

**Conclusions:**

The indication for this type of treatment must be selected and narrowed down, as it is a salvage procedure, and any failure would cause even more complex situations. Short operating times and early mobilization of the elbow are the advantages of this technique.

## Background

Managing distal humeral fractures (DHF) can be challenging for orthopedic surgeons. DHF has an incidence of 5.7 per 100.000 persons per year in adults and represent approximately 30% of fractures near the elbow [1].

Based on age and sex, these fractures have a bimodal distribution, occurring in young males following a high-energy trauma and in elderly women after low-energy falls [2].

DHF often happens after a fall with the elbow in a high degree of flexion (more than 110°) [3].

Radiographic evaluation should include anteroposterior, oblique, and lateral views of the elbow. Computed tomography (CT) scans are mandatory in fractures requiring surgery, and they are the key to planning surgery. The Orthopaedic Trauma Association (OTA/AO) classification is the most widely used classification system, in which these fractures are classified as 13C2 and 13C3 [4].

There are several treatment options for managing this type of fracture, and the treatment method for these fractures should be based on patient-related factors such as age, bone quality, comorbidities, and activity level before the injury [5]. In addition, the biomechanical complexity of the elbow articulation, the poor soft tissue coverage, and the proximity to functionally essential nerves and vessels add difficulties to the surgical procedure [6].

In young patients with good bone quality, the standard of care in comminuted DHF is double-plate osteosynthesis [7].

Instead, in cases of elderly patients with osteoporotic bone and severe comminution of the fracture, adequate fixation can be a major challenge for surgeons [8].

In these frail patients, severe comorbidities often make them ineligible for surgery, so that conservative treatment may be the only viable treatment option [9].

Instead, in those eligible for surgery, open reduction and internal fixation (ORIF) treatment remains the best option, but when ORIF is not possible, prosthetic elbow replacement can provide an early postoperative mobilization that is crucial in these patients [5].

Conventional total elbow arthroplasty (TEA) is an effective alternative to osteosynthesis that may offer good function and immediate mobilization in elderly low-demand patients with comminuted DHF and poor bone quality [10].

It is also the treatment of choice for patients with preexisting osteoarthritis of the elbow who sustain DHF. Disadvantages of TEA include lifelong weight restrictions to the extremities and risks of prosthetic loosening, fracture, infection, and poor longevity [1].

In case of severe bone loss, significant comminution, metaphyseal extension of the fracture, or in revision surgery of osteosynthesis or failure of prosthetic implants, different types of reconstruction of the elbow joint are described in the literature, ranging from osteoarticular allografts and Allograft-Prosthetic Composites (APC) to custom-made or modular megaprosthesis or arthrodesis [11–13].

Osteoarticular allografts and APCs are effective treatment options in these cases but are still associated with high complication rates [14].

Arthrodesis of the elbow joint significantly reduces pain, but the functional limitations in daily life are essential [15].

Modular or custom-made elbow megaprostheses may be a valuable option in large bone defects of the distal humerus in elderly patients, avoiding the structural problems of allograft reconstructions [16].

Megaprostheses are known, reliable, and effective reconstruction tools in oncologic surgery used for limb salvage in patients affected by primary or secondary bone or soft tissue tumors [17].

Megaprosthesis has been recently proposed in traumatology as an alternative to osteosynthesis or conventional prosthesis for managing comminuted articular fractures in elderly patients with poor bone stock [18–20].

The present study aimed to evaluate the clinical and functional outcomes of megaprosthetic implants in the treatment of comminuted distal humeral fractures (AO OTA 13C2 and 13C3) with metaphyseal extension in elderly patients with poor bone quality at a minimum of one year of follow-up.

## Materials and methods

A consecutive case series of 5 patients who underwent reconstruction of the elbow joint with a trabecular hinged modular elbow megaprosthesis between 2017 and 2022 were reviewed retrospectively. All patients included had AO/OTA 13C2 and 13C3 fractures with metaphyseal extension and considerable bone loss of the distal humerus.

### Study setting and design

A retrospective observational study according to the PROCESS guidelines was conducted on five patients with distal humeral fractures with metaphyseal extension and considerable bone loss of the distal humerus treated with trabecular hinged modular elbow megaprosthesis at our University Hospital between January 2017 and January 2022 [21]. The study respects national ethical standards and the Declaration of Helsinki. Written informed consent for surgical and clinical data collection for scientific purposes was obtained from all patients at the admission and before surgery according to institutional protocol.

### Inclusion and exclusion criteria

Inclusion criteria were: (I) distal epiphyseal multifragmentary humeral fractures with metaphyseal extension (AO classification 13C2 and 13C3); (II) considerable bone loss of the distal humerus that excluded the use of standard prostheses (III) use of trabecular hinged modular elbow megaprosthesis; (IV) the consent of the patient to be included in the study.

Exclusion criteria were: (I) a follow-up of less than one year, (II) oncological diseases, (III) open fractures, and (IV) incomplete radiological and clinical data set.

### Perioperative management

All patients underwent preoperative radiography and high-resolution CT (1 mm thin layer) of the affected segment. All prostheses were trabecular hinged modular megaprosthesis manufactured by the same company (Mutars Implantcast Ltd., Buxtehude, Germany). All of them were silver-coated to reduce infectious complications [22].

The same surgeon, oncologic, orthopedic, and traumatology ex, performed all the procedures. Preoperative antibiotic prophylaxis, with intravenous Cefazolin 2 g, was administered to all patients, per protocol in our institution, without penicillin allergy [23].

All patients underwent general anesthesia with orotracheal intubation. A urinary catheter was placed preoperatively. A tourniquet was applied to the proximal upper extremity, which was not inflated.

A supine position and an anterior surgical approach to the elbow prolonged proximally and laterally were performed in all resections.

At first, the lateral cutaneous nerve of the forearm was identified superficially to the fascia and protected for the duration of the surgery. The fascia was incised along the medial edge of the brachioradialis muscle.

The radial artery is identified under the bicipital aponeurosis and protected for the duration of the surgery. The radial nerve was identified and preserved between the brachialis and brachioradialis muscles and followed proximally until it passed through the lateral intermuscular septum.

After the osteotomy of the distal humerus and the mobilization of the surrounding tissues, the distal humeral resection was completed, protecting the ulnar nerve.

**Fig. 1:**
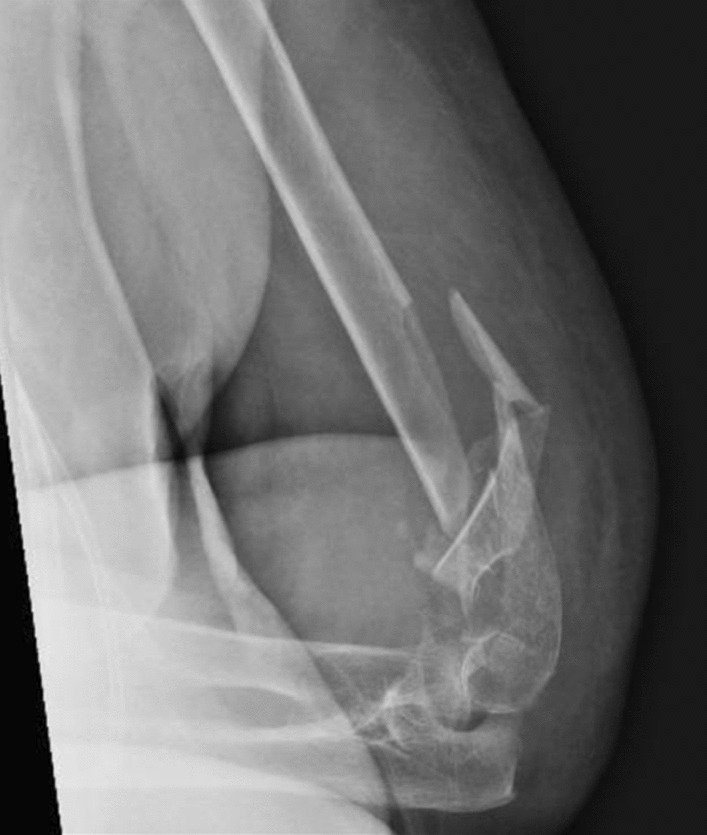
Preoperative X-ray

**Fig. 2:**
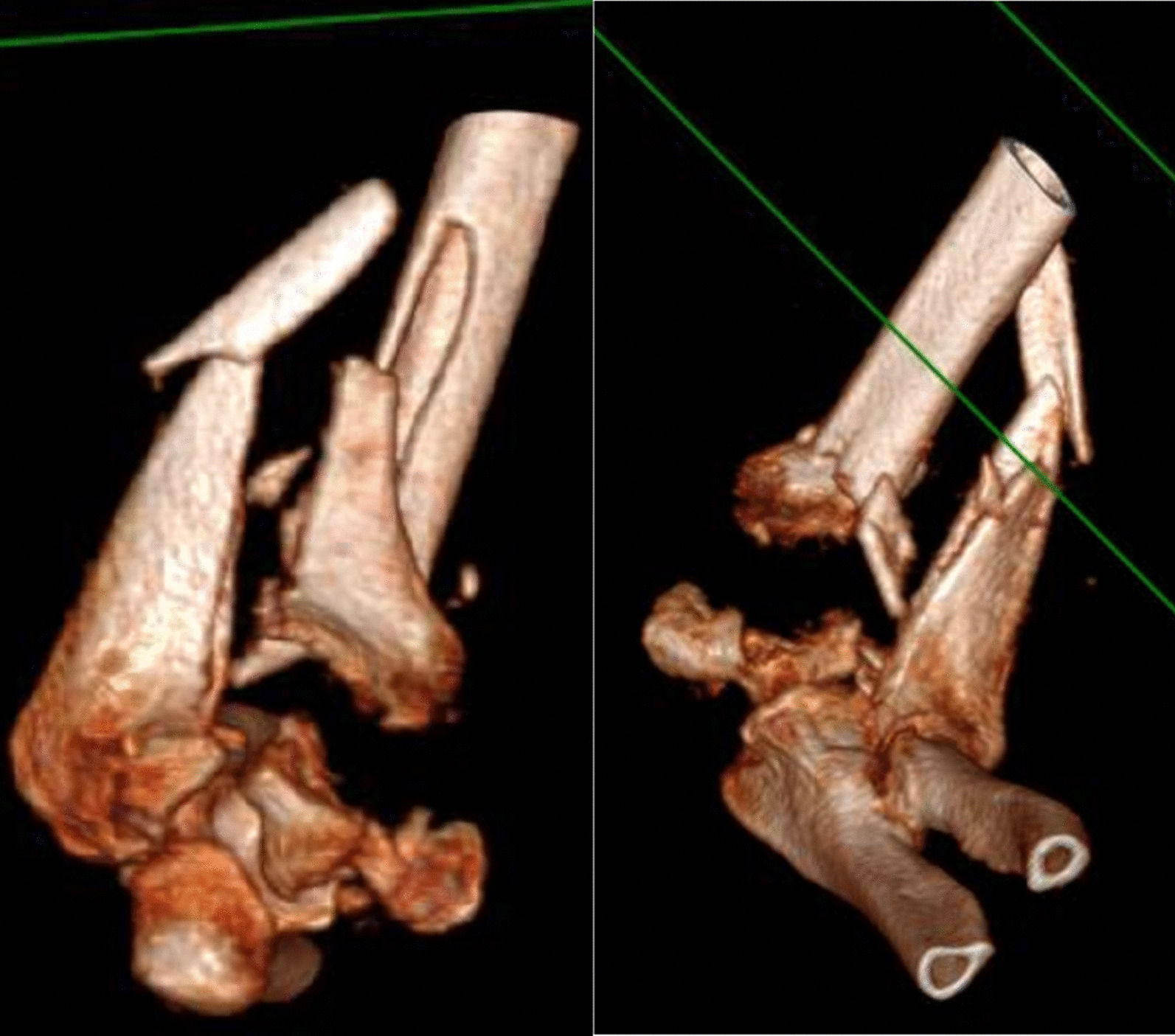
Preoperative CT scan, 3D bone reconstructions

**Fig. 3:**
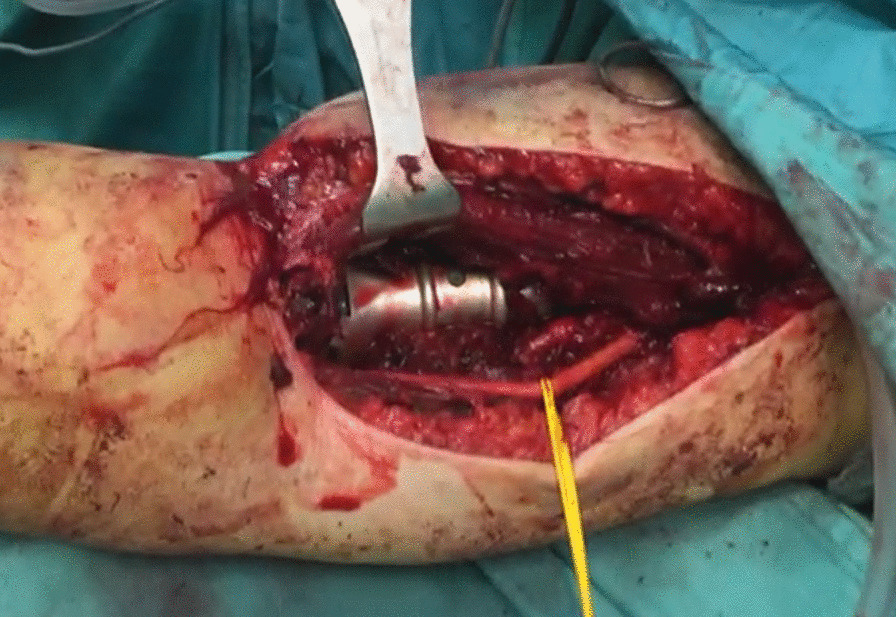
Intra-operative image with radial nerve neurolysis

**Fig. 4:**
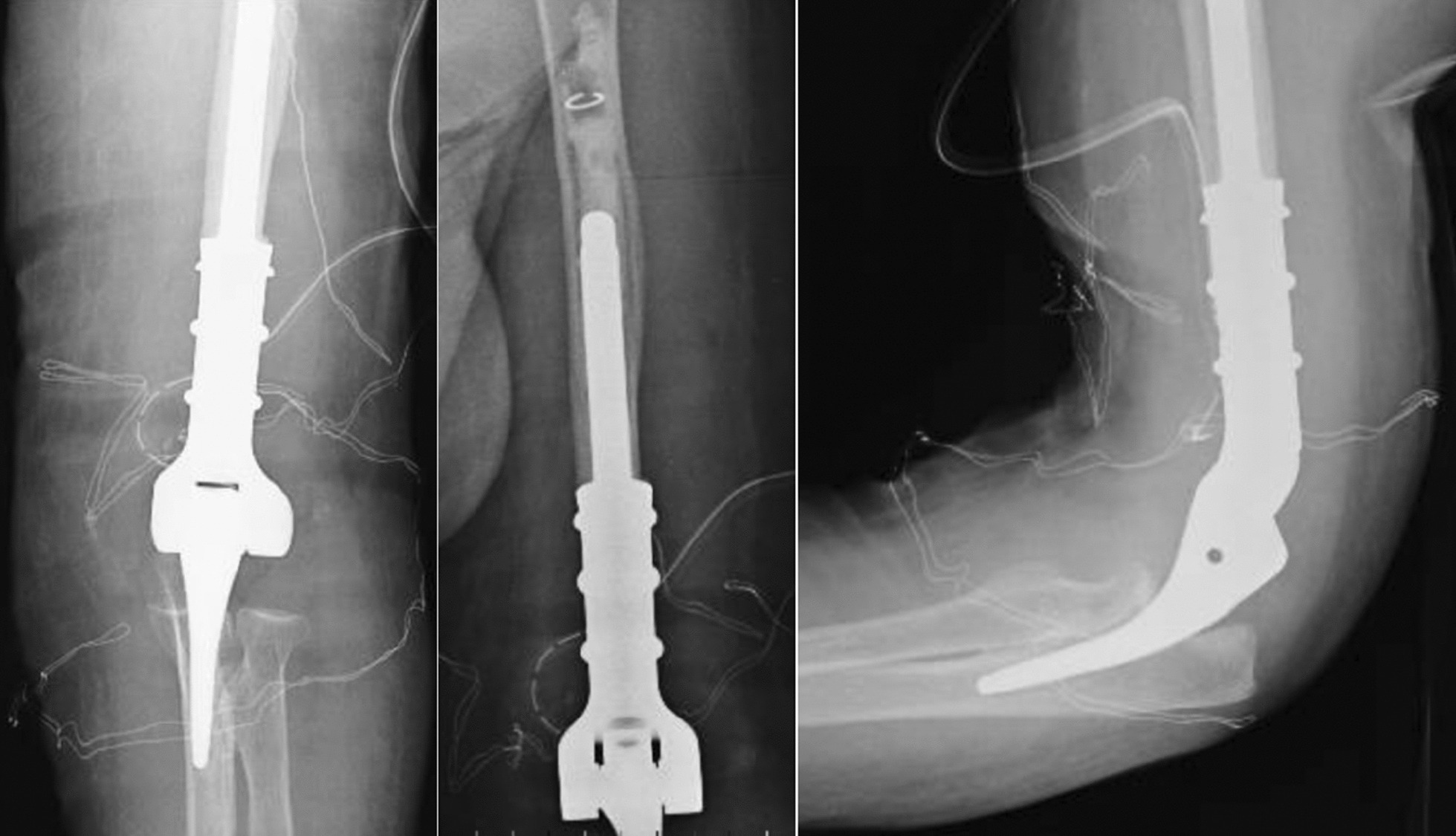
Post-operative X-ray

The level of humeral osteotomy was chosen based on each patient's fracture. Once the megaprosthesis was implanted, we performed intraoperative tests to evaluate the implant's stability, which was satisfactory in all cases. In all cases, an immediate postoperative x-ray in the operatory room was performed to confirm proper implant placement. Of 5 elbow megaprostheses, four were uncemented, and in only one case did we decide to cement the humeral component due to the patient's very poor humeral bone stock. We did not perform a resection of the radial head in these cases. (Figs. 1,2,3,4).

After the surgical procedure, a drainage tube was placed in all patients. Depending on the patient's bleeding, the surgical drainage tube was removed 24 or 48 h after surgery, while the urinary catheter one day after surgery. Immobilization with an arm brace with a chest strap was performed in all patients, passive elbow mobilization was allowed immediately after surgery, and active physiotherapeutic training was started after complete wound healing.

### Clinical radiological follow-up and complications

Each patient was systematically clinically and radiologically monitored at one, three, six, and twelve months after surgery and then once a year.

The Mayo Elbow Performance Score (MEPS) was used to evaluate clinical and functional outcomes during the follow-up. The MEPS is widely used for the evaluation of clinical outcomes for a variety of elbow disorders. This scoring system assesses pain, arc of motion, stability, and a patient rating of daily function. Pain is the weighted highest of the four variables. The scale ranges from 0 to 100, and the outcome is rated as follows: excellent, 90–100 points; good, 75–89 points; fair, 60–74 points; or poor, less than 60 points [24].

Complications were considered intraoperative (damage or palsy of a nerve, vascular iatrogenic lesions, intraoperative fractures) and postoperative, in turn, divided into early (within 6 postoperative months) and late (more than 6 months after surgery). The postoperative complications evaluated were prosthetic joint infections (PJI), aseptic loosening, periprosthetic fractures, and surgical site infections (SSIs).

For diagnosing periprosthetic infection, we used the criteria of the Musculoskeletal Infection Society (MSIS) 2011 [25].

Aseptic loosening is the failure of joint implants without the presence of a mechanical cause or infection. It is defined based on X-ray evidence [26].

Periprosthetic fractures are considered fractures associated with an orthopedic implant, whether a replacement or internal fixation device [27].

The ECDC defines SSIs as microbial contamination of the surgical wound within 30 days of an operation or 1 year after surgery if an implant is placed in a patient [28].

### Outcomes

The primary outcome was the evaluation of functional and clinical outcomes with the MEPS score in comminuted distal humerus fractures with metaphyseal extension and poor bone stock in elderly patients treated with elbow megaprosthesis. The secondary outcome was assessing the treatment-related complication rate of this technique in non-oncological fields.

## Results

Five patients were included in the study, four females and one male, with a mean age of 82.66 ± 7.72 years at surgery. The fractures were distal epiphyseal multifragmentary humeral fractures with metaphyseal extension and poor bone stock (Table 1). Once the CT examination was performed, we classified these five fractures as AO/OTA classification four as 13C3 and one as 13C2.

**Table 1:** Demographics data and patients charateristics

Number of patients	5
Gender	1 M
	4 F
Average age (yo)	82.66 ± 7.72
AO Classification Fracture	4 11C3
	1 11C2
Hb preoperative (mg/dL)	11.66 ± 1.59
Hb post-operative 1POD (mg/dL)	9.4 ± 0.75
Average stay (days)	7.3 ± 2.49
Follow-up (months)	17.2 ± 4.66

*M*: male; *F*: female; *Hb*: hemoglobin; *POD*: post-operative day; *yo*: years old

The mean preoperative hemoglobin value was 11.66 mg/dL ± 1.59. The mean postoperative hemoglobin value was 9.4 mg/dL ± 0.75.

The mean length of hospitalization was 7.3 days ± 2.49, and the mean follow-up was 17.2 months ± 4.66.

The mean MEPS value was 63 ± 24.2 at 1 month, 81 ± 23.53 at 3 months, 83 ± 24.2 at 6 months, and 84 ± 24.57 at 12 months.

No intraoperative complications were recorded in our series. Of 5 patients, four patients had excellent clinical and functional outcomes. The last patient had a radial nerve injury caused by trauma. Radial nerve deficit was clinically detected before surgery. Intraoperatively, however, the radial nerve appeared to be imprisoned at the fracture site without conspicuously visible lesions. She had postoperative persistent pain and ROM limitations, and the radial nerve deficit had only partially recovered at the last assessment at 12 months of follow-up, with MEPS scores worse than the other patients.

The patient was followed in postoperative period with electromyography every 3 months. She has been treated with physiotherapy, electrical stimulation and neurotrophic drugs. 2 years later the patient wears a radial nerve splint and she is not satisfied. Today the electromyography exam is stable with the absence of functional recovery. In the last visit, we have proposed a tendon transfer as possible option to supply the radial nerve injury.

We did not encounter wound dehiscence, prosthetic joint infection, aseptic loosening, or periprosthetic fractures.

**Fig. 5:**
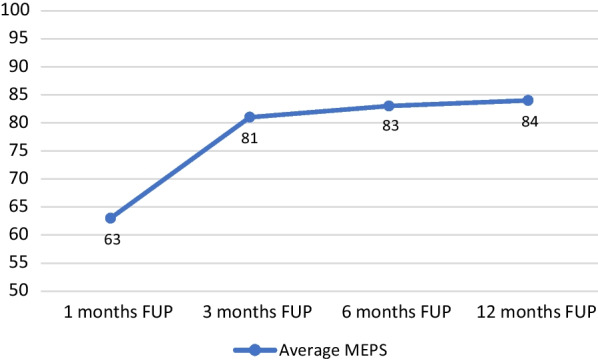
MEPS: Mayo elbow performance score; FUP: Follow up

The MEPS results the patients underwent at 1, 3, 6, and 12 months after surgery were collected and summarized in Fig. 5.

## Discussion

Comminuted distal humerus fractures in elderly patients with poor bone stock are surgically demanding situations. The biomechanical complexity of the elbow joint, the poor soft tissue coverage, and the proximity to vital neurovascular structures make it a major challenge for the orthopedic surgeon [8].

Furthermore, in elderly patients, it is crucial to choose a treatment method that provides stability and allows early postoperative mobilization, thus minimizing the risk of associated medical complications [6].

In these complex situations, the surgical treatment options may include osteoarticular allografts, APC, arthrodesis, and custom-made and modular megaprosthesis but also conservative treatment is to be considered.

Regarding the outcomes of osteoarticular allografts, Kharrazi et al. in 2008 reported 19 cases with a 32% complication rate, including nonunion, infection, and allograft resorption [11].

Laumoniere et al. (2022) described using APCs in treating eight patients with aseptic loosening of TEA with massive humeral bone loss. The authors reported complications in 5 patients in 8 recruited [29].

Elbow arthrodesis is supposed to be “a procedure that is unsatisfying for both the patient and the surgeon and should be retained as an exception” [30].

In addition to having poor functional outcomes, elbow arthrodesis is not a procedure without complications. Koller et al. reported a 43% complication rate in 14 patients undergoing elbow arthrodesis [15].

According to a recent work by Loisel et al. of 2023 the conservative treatment for distal humerus fractures is still a viable alternative treatment with high rate of complications and non-unique outcomes; nevertheless it should be considered in elderly patients who cannot undergo surgical treatment [31].

The use of megaprostheses is a viable alternative in cases where bone stock at the fracture site is so severely compromised that traditional internal fixation or joint replacement would not be enough to provide the needed stability to allow early mobilization and durable longevity of the implant [32].

Megaprosthesis started to appear in the 1940s as a limb salvage treatment for bone defects caused by osteosarcoma [33].

Indications for the use of megaprosthesis are still a matter of discussion. The main indication remains the massive bone loss after large bone tumor excision around the joint. However, some authors support their use in cases of poor bone quality, which renders it impossible to use other surgical procedures [32].

Megaprosthesis has been recently proposed in traumatology as an alternative to osteosynthesis or conventional prosthesis replacement for managing comminuted articular fractures in elderly patients with poor bone stock, especially in the lower limb [34].

Once considering the good clinical and functional outcomes obtained with megaprosthesis implantation in comminuted hip and knee fractures, we also proposed this treatment for comminuted distal humeral fractures with metaphyseal extension in elderly patients with poor bone quality [12].

Reports from the literature about patients with primary reconstruction with modular megaprosthesis after severely comminuted fracture or massive bone loss are rare, but the results of mid-term follow-up studies on this topic seem to be encouraging.

Ziranu et al. reported a mean implant survival rate of 85% at one year and 82% at two years in 36 oncological and non-oncological patients treated with proximal femur replacement. The authors concluded that megaprosthesis can also be a valid treatment option in non-oncological cases, specifying that only selected cases should undergo this kind of surgical procedure because of the high complication rate [35].

Lundh et al. reported an overall hip and knee megaprosthesis survival rate of 94% on 17 patients at 44 months of follow-up [32].

Even fewer studies regarding elbow megaprostheses in traumatology can be found in the literature.

In a recent case series, Trung et al. reported excellent clinical and function outcomes in 2 patients with non-oncologic conditions and severe bone loss at the elbow treated with custom-made megaprosthesis at a mean follow-up period of 14 months. They concluded that megaprosthesis elbow arthroplasty is a very reliable and practical choice in treating large bone defects around the elbow joint, helping to reduce the pain and rehabilitate the function of the elbow joint best [36].

Although this is an excellent option to help store shape and function in large bone defects, many authors have also reported the complication rate after megaprosthesis elbow arthroplasty.

In 2011, Funovics et al. reported 52 modular megaprosthesis in patients affected by osteosarcoma or very severe elbow osteoarthritis. In 34 patients, they did not find complications, while 18 patients needed revision surgery due to complications related to artificial joints, no wound healing, and radial and ulnar nerve palsy [12].

Capanna et al. in 2016 reported that of 36 patients, 31 were oncological patients, and 5 were revisions of previous prosthetic implants with massive bone loss; there were six complications. The authors also reported that the complication rate with elbow megaprosthesis is lower than that of other methods, such as bone graft and other composites, while the functional outcome is equal. Moreover, patients better accept this technique cosmetically and emotionally than elbow arthrodesis [20].

In our study, we have recruited five elderly patients with comminuted fractures of the distal humerus, with metaphyseal extension and massive bone loss, who are not eligible for ORIF or conventional TEA. Of these five patients, 4 had excellent clinical and functional results at least one year of follow-up. In contrast, one patient had persistent pain, ROM limitations, and radial nerve deficit due to the trauma only partially recovered at the last assessment at 12 months of follow-up. In any case, despite the poor functional scores of this unfortunate patient, a mean MEPS score of 84 at 12 months of follow-up indicates good clinical and functional outcomes.

It should also be emphasized that we did not find any intra- or postoperative complications in our patients at least one year after follow-up [37].

Few authors in the literature have described the use of elbow megaprosthesis as the primary treatment for comminuted distal humerus fractures with severe bone loss [36].

This did not allow us to compare our results with a large cohort of patients.

The advantages of treatment with megaprostheses are lower operating times, early postoperative mobilization, and good functional results. The disadvantages, on the other hand, are the restriction of the weights that can be lifted (no more than 5 kg) and the possible complications related to a prosthetic implant, therefore aseptic mobilizations, periprosthetic fractures, prosthetic infections, etc.

This study has several limitations. Firstly, the patient cohort is small; however, as this is a rarely performed treatment, it was impossible to have a more extensive study population with restricted inclusion criteria. Secondly, the study was a retrospective design and was not blinded, which causes potential bias and limits the strength of our conclusions. Finally, the follow-up periods are relatively short, so this study needs to assess the long-term effects of this type of treatment.

It should be noted that, to our knowledge, this study is the one with the largest case series in the literature on the treatment of DHF with elbow megaprosthesis in elderly patients.

## Conclusions

Megaprostheses are a valid treatment option in elderly patients with comminuted distal humerus fractures with metaphyseal extension and poor bone quality. The indication for this type of treatment must be selected and narrowed down, as it is a salvage procedure, and any failure would cause even more complex situations.

Megaprostheses are a one-shot surgery, with variable outcomes reported in the literature [34]^.^

Short operating times and early mobilization of the elbow are the advantages of this technique. Further studies are needed to confirm the validity of this treatment in this type of patient and fracture, possibly with longer follow-ups to evaluate the long-term effects of this procedure.

## Data Availability

The study data will be available upon request to the corresponding author (email: luigi_cianni@libero.it).

## Abbreviations

DHF: Distal humerus fractures
CT: Computer tomography
OTA: Orthopaedic trauma association
AO: Association of ostheosynhtesis
ORIF: Open reduction and internal fixation
TEA: Total elbow arthroplasty
APC: Allograft prosthetic composites
MEPS: Mayo elbow performance score
PJI: Prosthetic joint infections
SSIs: Surgical site infections
MSIS: Musculoskeletal infection society
Hb: Hemoglobin
POD: Postoperative day
YO: Years old
FUP: Follow-up
